# TNF Receptor 2 and Disease: Autoimmunity and Regenerative Medicine

**DOI:** 10.3389/fimmu.2013.00478

**Published:** 2013-12-23

**Authors:** Denise L. Faustman, Miriam Davis

**Affiliations:** ^1^Immunobiology Laboratory, Massachusetts General Hospital and Harvard Medical School, Boston, MA, USA; ^2^Immunobiology Laboratory, Massachusetts General Hospital, Boston, MA, USA

**Keywords:** TNF, TNF receptor 2, autoimmune disease, type 1 diabetes, regeneration

## Abstract

The regulatory cytokine tumor necrosis factor (TNF) exerts its effects through two receptors: TNFR1 and TNFR2. Defects in TNFR2 signaling are evident in a variety of autoimmune diseases. One new treatment strategy for autoimmune disease is selective destruction of autoreactive T cells by administration of TNF, TNF inducers, or TNFR2 agonism. A related strategy is to rely on TNFR2 agonism to induce T-regulatory cells (T_regs_) that suppress cytotoxic T cells. Targeting TNFR2 as a treatment strategy is likely superior to TNFR1 because of its more limited cellular distribution on T cells, subsets of neurons, and a few other cell types, whereas TNFR1 is expressed throughout the body. This review focuses on TNFR2 expression, structure, and signaling; TNFR2 signaling in autoimmune disease; treatment strategies targeting TNFR2 in autoimmunity; and the potential for TNFR2 to facilitate end organ regeneration.

## Introduction

Tumor necrosis factor (TNF) is a pleiotropic cytokine involved in regulating diverse bodily functions including cell growth modulation, inflammation, tumorigenesis, viral replication, septic shock, and autoimmunity (1). These functions hinge upon TNF’s binding to two distinct membrane receptors on target cells: TNFR1 (also known as p55 and TNFRSF1A) and TNFR2 (also known as p75 and TNFRSF1B). TNFR1 is ubiquitously expressed on the lymphoid system and nearly all cells of the body, which likely accounts for TNF’s wide-ranging functions. TNFR2 is expressed in a more limited manner on certain populations of lymphocytes, including T-regulatory cells (T_regs_) (2, 3), endothelial cells, microglia, neuron subtypes (4, 5), oligodendrocytes (6, 7), cardiac myocytes (8), thymocytes (9, 10), islets of Langerhans (personal communication, Faustman Lab), and human mesenchymal stem cells (11). Its more restricted cellular expression makes TNFR2 more attractive than TNFR1 as a molecular target for drug development. Activation of TNFR1 alone by exogenous TNF is systemically toxic (12, 13).

As a general rule, TNF depends on TNFR1 for apoptosis and TNFR2 for any function related to cell survival, although there is some degree of overlapping function depending upon the activation state of the cell and a variety of other factors (14). Likewise, TNFR1 and TNFR2 have distinct intracellular signaling pathways, although there is some overlap and crosstalk (15). TNF binding to TNFR1 triggers apoptosis through two pathways, by activation of the adaptor proteins TNFR1-associated death domain (TRADD) and Fas-associated death domain (FADD). In contrast, TNFR2 signaling relies on TRAF2 and activation and nuclear entry of the pro-survival transcription factor nuclear factor-kB (NFkB) (16–18). TNFR2 expression on T_regs_ is induced upon T-cell receptor activation (19).

While the etiologies of autoimmune disorders vary, there is some degree of overlap in their genetic, post-translational, and environmental origins. One overlapping feature is that various defects in TNF signaling pathways, acting through the TNF receptors and NFkB in autoreactive T cells, occur in both human and mouse models of various autoimmune disorders, including Crohn’s disease, Sjogren’s syndrome, multiple sclerosis, ankylosing spondylitis, and type I diabetes (20–39). The defects range from defects in the proteasome in both the non-obese diabetic (NOD) mouse model and humans with Sjogren’s syndrome, to specific polymorphisms in the TNFR1 or TNFR2 receptors themselves, to punitive interruptions in genes that control the ubiquitination of the NFkB pathway.

## TNFR Expression, Structure, and Signaling

As noted above, TNFR1 and TNFR2 possess different patterns of expression. TNFR1 is found on nearly all bodily cells, whereas TNFR2 is largely found on certain immune cells (CD4+ and CD9+ lymphocytes), certain CNS cells, and endothelial cells, among others. Neither receptor is located on erythrocytes. Typically, cells that express TNFR2 also express TNFR1, with the ratio of expression varying according to cell type and functional role. Because TNFR1 typically signals cell death, while TNFR2 typically signals cell survival, the ratio of their co-expression will shift the balance between cellular survival and apoptosis.

**Figure 1:**
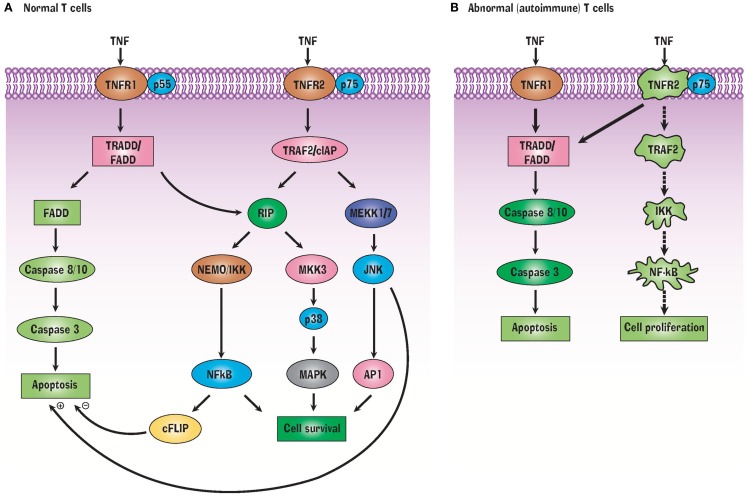
**TNF Signals through TNFR1 and TNFR2 receptors (A) but abnormalities in this signaling pathway in autoimmunity (B) can favor a pathway of selective apoptosis due to a variety of protein signaling defects**.

TNFR1 and TNFR2 have extracellular, transmembrane, and cytoplasmic components. The extracellular component of both receptors is rich in cysteine, which is characteristic of the TNF superfamily. TNFR1 contains 434 amino acids. Its intracellular region of 221 amino acids contains a death domain that binds TRADD or FADD. In T cells, activation of TRADD or FADD activates the caspases, resulting in apoptosis (Figure 1). A second apoptotic pathway relies on TRADD’s activation of RIP (receptor interacting protein) (Figure 1A). In contrast to TNFR1, TNFR2 does not have a cytoplasmic death domain. The receptor consists of 439 amino acids. Its extracellular domain is formed by the first 235 amino acids, its transmembrane domain is formed by 30 amino acids, while its cytoplasmic domain is formed by 174 amino acids. TNFR2’s cytoplasmic domain has a TRAF2 binding site. TRAF2, in turn, binds TRAF1, TRAF3, cIAP1, and cIAP2 (17, 18). These signaling proteins activate several other signaling proteins, yielding cell survival (Figure 1A). Cell survival is ensured when the transcription factor NFkB is liberated from its inhibitor protein IkBα in the cytoplasm and translocates to the nucleus where it activates pro-survival target genes (40). Both TNFR1 and TNFR2 can bind monomeric TNF or trimeric soluble TNF although soluble TNF induces no or weak signaling for TNFR2. This may be related to altered association or dissociation kinetics or more optimal kinetics with pre-formed transmembrane TNF (41). TNFR2 also preferentially binds transmembrane TNF (42). Transmembrane TNF is a trimer on the cell surface and transmits signals to the cell where it is contained, i.e., reverse signaling. It is thought that TNFR2 preferentially binds transmembrane bound TNF (43). Solution of the crystal structure of the TNF-TNFR2 complexes demonstrated that these interactions also result in the formation of aggregates on the cell surface and this likely promotes signaling (44).

Transgenic mice have been produced to try to understand better the function of TNFR2 (45). TNFR2^−/−^ mice homozygous for TNFR2^−/−^ are viable and fertile. They also show normal T-cell development and activity and are resistant to TNF-induced death. The T-cell proliferation responses are diminished and they also show abnormal central nervous system regeneration (JAX Mice Database – 00260).

## TNF in Development and Autoimmunity

Tumor necrosis factor, and its signaling through the two receptors, plays several crucial roles during normal development. It shapes the efficacy of the immune system and protects against infectious disease, cancer, and autoimmune disease (46). Upon release, TNF proceeds throughout the lifecycle to exert regulatory roles over immune cells by triggering transcription of genes responsible for inflammation, proliferation, differentiation, and apoptosis. To counter a pathological infection, TNF facilitates proliferation of immune cell clones. To continue to fight against the infection, TNF stimulates differentiation and recruitment of naïve immune cells. Subsequently, TNF orchestrates destruction of superfluous immune cell clones to reduce inflammation and tissue damage once the infection is resolved.

In the process of developing autoimmunity, abnormal progenitors to T cells and other immune cell types proliferate and begin to mature in the thymus. T-cell education occurs through two parallel pathways for CD4 and CD8 T cells through either HLA class II or HLA class I cell surface structures. For almost all autoimmune disease there is strong genetic linkage to the HLA class II region. This genetic region is rich in immune response genes and contains not only the class II genes themselves but also the HLA class I assembly genes such as the tap transporters (Tap1/Tap2) and proteasome genes (that control self peptide presentation) such as LMP2 (PSMB9), LMP7 (PSMB8), and LMP10 (PSMB10) (47). During T-cell education, the vast majority of immature immune cells die by apoptosis, which serves to remove defective progenitors. The process is not foolproof, however. Failures in T-cell education in humans perhaps driven by defective antigen presentation allow autoreactive but still immature T cells defined as CD45RA+ (2H4) and lesser numbers of CD45RO+ (4B4) to enter the circulation (36, 48, 49). In humans and autoimmune animal models diverse mutations and polymorphisms drive altered proteasome function with varying phenotypes of autoimmunity (50–55). Once in the circulation, the cells differentiate into mature autoreactive T cells when they encounter specific self-antigens (56). The failure of T-cell education of autoreactive CD8 T cells, due to HLA class I interruption, yields self-reactive T cells directed at specific self-antigens. This failure underlies various immune diseases, including type I diabetes, Crohn’s disease, multiple sclerosis, and Sjogren’s syndrome (50).

## TNFR2 Signaling and Benefits to Health

TNFR2 signaling pathways appear to offer protective roles in several disorders, including autoimmune disease, heart disease, demyelinating and neurodegenerative disorders, and infectious disease. According to *in vitro* and *in vivo* studies, TNF or TNFR2 agonism is associated with pancreatic regeneration (57–59), cardioprotection (60, 61), remyelination (5, 6), survival of some neuron subtypes (5, 62, 63), and stem cell proliferation (11, 64–66).

Knockout of the *tnfr2* gene in a mouse model produces a higher rate of heart failure and reduced survival after myocardial infarction (60). TNFR1 signaling is deleterious and TNFR2 signaling is protective in regeneration and repair processes following infarcted myocardium in female mice (61).

An agonist for TNFR2 selectively destroys autoreactive T cells but not healthy T cells in blood samples from type I diabetes patients, as well as multiple sclerosis, Graves, Sjogren’s autoreactive T cells (57). Animal models of type I diabetes exhibit massive regeneration of the pancreas after elimination of autoreactive T cells with low-dose TNF (58, 59). TNFR2 is crucial for TNF-induced regeneration of oligodendrocyte precursors that make up myelin (6), a finding that may be important in the treatment of multiple sclerosis and other demyelinating disorders, regardless of whether they have an autoimmune etiology. In viral encephalitis-infected knockout mice, the TNFR2 pathway is relied upon to repair the brain’s hippocampus, and TNFR1 is relied upon to repair the brain’s striatum (63). Oligodendrocyte regeneration appears to occur as a result of TNFR2 activation on astrocytes, which promotes oligodendrocyte proliferation through the induction of chemokine CXCL12 in an animal model of demyelination (67). Lastly, TNFR1 promotes neurodegeneration while TNFR2 promotes neuroprotection in an animal model of retinal ischemia in knockout mice (68).

## TNF Receptor and Autoimmune Disease

A variety of defects in TNFR2 and downstream NFKB signaling are found in various autoimmune diseases. The defects include polymorphisms in the TNFR2 gene, upregulated expression of TNFR2, and TNFR2 receptor shedding. A recently published study implicates a new decoy splice variant of the TNFR1 receptor in multiple sclerosis. This causes a relative deficiency in TNF with inadequate TNFR2 signaling for autoreactive T-cell selection and induction of beneficial T_regs_ (39). Polymorphisms in TNFR2 have been identified in some patients with familial rheumatoid arthritis (69–71), Crohn’s disease (72), ankylosing spondylitis (38), ulcerative colitis (73), and immune-related conditions such as graft versus host disease associated with scleroderma risk (74). Common to several autoimmune diseases, with the notable exception of type I diabetes, is a polymorphism in which the amino acid methionine is substituted for arginine at position 196 in exon 6 of chromosome 1p36 (16). This polymorphism may alter the binding kinetics between TNF and TNFR2, the result of which may reduce signaling through NFkB.

Upregulated expression of TNFR2 is also found in several immune diseases (16, 75). Higher systemic levels of soluble TNFR1 (sTNFR1) and soluble TNFR2 (sTNFR2) are produced by administration of TNF to patients, likely by shedding of receptors into the extracellular space (76, 77). The greater the TNF stimulation, the greater is the increase in sTNFR1 and sTNFR2. Higher levels of sTNFR2 but not sTNFR1 are found in serum and bodily fluids of patients with familial rheumatoid arthritis (78) and systemic lupus erythematosus, both of which are marked by polymorphisms in TNFR2. TNFR2, but not TNFR1, is upregulated in the lamina propria of mice with Crohn’s disease, and it causes *in vivo* experimental colitis (79). Decreasing the concentration of TNFR2, via receptor shedding or other means, is a possible compensatory mechanism to lower inflammation. The extracellular component of TNFR2 is proteolytically cleaved to produce sTNFR2. This component binds to TNF in the extracellular space, yielding lower concentrations of TNF available for binding to functional T cells (80, 81). The development of the first anti-TNF medications, including soluble TNFR2 fusion proteins like Enbrel, were therapeutic for some patients with rheumatoid arthritis but consistently worsened or induced new autoimmune diseases like type 1 diabetes, lupus, or multiple sclerosis. The human data are consistent with past mouse data where overexpression of TNFR2 triggered multi-organ inflammation especially in the presence of TNF.

To achieve cell survival, the final steps in the TNFR2 pathway rely on NFkB mobilization and translocation to the nucleus. This can only occur with an intact proteasome, which is responsible for cleaving the bond between NFkB and its inhibitor protein IKBA. A defect that inhibits proteasomal-driven cleavage of NFkB is seen in the type I diabetes-prone and Sjogren’s syndrome-prone NOD mouse (33). A protein subunit of the proteasome, LMP2, is lowered in all patients with Sjogren’s syndrome (36, 52, 82). The LMP2 subunit of the proteasome is necessary for intracellular activation of NFkB in highly activated T cells (33).

## TNF as Treatment for Autoimmune Disease

Given the commonality of TNFR signaling abnormalities in autoimmune diseases, the administration of TNF has emerged as a common treatment strategy. Low-dose TNF exposure, acting through its receptors, selectively destroys autoreactive, but not healthy, CD8+ T cells in blood samples from patients with type I diabetes (57). Low-dose TNF also kills autoreactive T cells in an animal model of Sjogren’s syndrome (83). A similar result with TNF exposure is achieved in blood samples from patients with scleroderma (84). A sustained effect need not require continuous dosing, unlike treatment with anti-cytokines or immunosuppressive drugs: TNF can be effective when administered intermittently (33). However, the administration of TNF is not feasible in humans because it is systemically toxic when given to cancer patients who already have high TNF levels due to an intrinsic defense system (12, 13, 85). The toxicity of TNF likely stems from the ubiquitous cellular expression of TNFR1. Because TNFR2 is more restricted in its cellular expression, TNFR2 agonism may offer a safer therapeutic approach than administration of TNF. The possibility of intermittent exposure would also enhance the safety profile. As noted earlier, upregulated expression of TNFR2 in the target tissue is observed in several autoimmune disorders on the target; this target tissue expression may be responsible for the growth-promoting and regenerative properties of TNF agonism. In a baboon study, TNFR2 agonism was generally safe but exhibited adverse effects in the form of thymocyte proliferation, a febrile reaction, and a small, transient inflammation caused by mononuclear cell infiltration (86). Not all TNFR2 antibodies are the same, however, as some can bind to the receptor without eliciting an immune response. It may well be the case that tissue-specific or cell-specific therapies afford a better safety profile. Many factors have profound effects on the nature of TNFR signaling with antibody agonists. Their safety and efficacy are affected by changes in the ligand, receptor, adapter proteins, or other members of the signaling pathway. Findings may also vary depending on culture conditions, origin of cells, and activation state.

The rationale for TNFR2 agonism as therapy for autoimmune disease was first shown in type I diabetes. TNFR2 agonism or induction of TNF is an effective means of selectively killing autoreactive CD8+ T cells in animal models, in human cells *in vitro* (33, 58, 83, 87, 88) and in blood samples taken from patients with type I diabetes (57). In the latter study, there was a dose-response relationship between TNFR2 agonism and CD8+ T-cell toxicity. The CD8+ T cells were autoreactive to insulin, a major autoantigen in type I diabetes.

How is TNF effective at killing autoreactive T cells? A variety of TNFR2 signaling defects prevent liberation of NFkB from IkB, precluding transcription of pro-survival genes. This in turn biases autoreactive T cells to shift to the TRADD/FADD cell death signaling pathway which leads to apoptosis (Figure 1B). In other words, NFkB dysregulation makes autoreactive T cells selectively vulnerable to TNF-induced apoptosis (20). T cells, unlike B cells and other immune cells, do not constitutively express the active form of NFkB. Only this active form can translocate to the nucleus in order to transcribe pro-survival genes.

## Therapeutic Strategies for Autoimmune Disease

### Small-molecule agonists

Medicinal chemists have found it challenging to create receptor-specific agonists for the TNF superfamily. Developing an antagonist is generally accomplished more readily than developing an agonist. That said, peptides, antibodies, and small molecules have been developed as TNFR2 agonists (89, 90). Of these types, antibody agonists have been more effective at engaging a specific signaling pathway (57). In a labor-intensive process, TNFR2 agonists have been developed by point mutations in the TNF protein by site-directed mutagenesis (90). Our laboratory has recently generated a TNFR2 agonist that activates TNF signaling pathways and suppresses CD8 T cells (91). The advantage of this agonist is that it also induced proliferation of T_reg_ cells that exert an immunosuppressive function. TNFR2 agonists, while less toxic than TNFR1 agonists, still may have toxicities, especially to cells within the CNS (16). For that reason it may be desirable to develop agonists that do not succeed at crossing the blood-brain barrier.

### TNF inducers

The foremost inducer of TNF is the mycobacterium bovis bacillus Calmette–Guerin (BCG), which has been on the market for decades as a vaccine for tuberculosis and as a treatment for bladder cancer. Its chemical equivalent that does not meet FDA’s standards for purity is complete Freund’s adjuvant (CFA). In an early double blinded placebo-controlled Phase I clinical trial, BCG administration produced a transient increase in TNF in the circulation (92). BCG or CFA have been successfully used in animal models of type I diabetes to either prevent onset of diabetes or kill autoreactive T cells, leading to the restoration of pancreatic islet cell function and normoglycemia (58, 59, 93–95). Furthermore, in a proof-of-concept randomized, controlled clinical trial, BCG killed the insulin-autoreactive T cells in the circulation of patients with type I diabetes (92). With the removal of insulin-autoreactive T cells, pancreatic islets managed to regenerate to the extent that there was a transient rise in C-peptide, a marker for insulin production. The transient rise in C-peptide was striking, considering that patients in the trial averaged 15 years of disease. This clinical trial data repudiated the presumption that loss of pancreatic function is irreversible. Although BCG and CFA release TNF and therefore are not specific for TNFR2, they have low toxicity and thereby may be safe for treating autoimmune disease by virtue of inducing low levels of TNF.

### NFkB pathway modulation

Nuclear factor-kB is thwarted from entering the nucleus to transcribe pro-survival genes in autoimmune diseases featuring defects in TNF signaling (33, 34). Instead of being cleaved, NFkB remains bound in the cytoplasm to its inhibitory chaperone protein IkBa. A genetic defect in type I diabetes-prone and Sjogren’s syndrome-prone NOD mouse blocks the proteasome from cleaving NFKB from IkBa (34). Patients with Sjogren’s syndrome also exhibit this defect (52). Consequently, inhibiting NFkB’s translocation to the nucleus offers another therapeutic approach to autoimmune disease if it could be done in the select cells that are disease causing.

### TNFR1 antagonism

Tumor necrosis factor binds to TNFR1 and TNR2. Another way to make TNF selective for TNFR2 signaling, an effect that could promote tissue regeneration and remove autoimmunity, is to create a TNFR1 antagonist. This strategy would bias TNF to act solely through the TNFR2 receptor. This strategy also appears promising for hepatitis or autoimmunity in murine models (96). A humanized TNFR1-specific antagonistic antibody for selective inhibition of TNF action has been tested with promising results (96–98).

### Expansion of T-regulatory cells via TNFR2

T-regulatory cells are a type of immunosuppressive cell that displays diverse clinical applications in transplantation, allergy, infectious disease, GVHD, autoimmunity, and cancer (99). T_regs_ co-express CD4+ and the interleukin-2 receptor alpha chain CD25 hi and feature inducible levels of intracellular transcription factor forkhead box P3 (FOXP3). Naturally occurring T_regs_ appear to express TNFR2 at a higher density than TNFR1 (3, 100, 101). There is evidence from animal models that TNF signaling through TNFR2 promotes T_reg_ activity: TNFR2 activates and induces proliferation of T_regs_ (100) and TNFR2 expression indicates maximally suppressive T_regs_ (102).

T-regulatory cells have been proposed to prevent or treat autoimmune disease, but the rate-limiting problem has been obtaining sufficient quantities, whether by generating them *ex vivo* or stimulating their production *in vivo*. *In vivo* stimulation turns out to be too toxic with standard expansion agents IL-2, anti-CD3, and anti-CD28. These expansion agents can be used to generate large quantities of T_regs_ *ex vivo*, but the problem is that they produce heterogeneous progeny consisting of mixed CD4+ populations. Heterogeneous progeny carry risk: they are capable of releasing pro-inflammatory cytokines and consist of cell populations with antagonistic properties. Some new approaches are being attempted, including expansion of T_regs_ *in vivo* with TL1A-Ig, a naturally occurring TNF receptor superfamily agonist (103). Additionally, our laboratory has developed a method of *ex vivo* expansion using a newly synthesized TNFR2 monoclonal antibody agonist that produces homogeneous progeny expressing a uniform phenotype of 14 cell surface markers (91). The TNFR2-agonist expanded T_regs_ are capable of suppressing CD8+ T cells. In healthy humans, the TNF inducer BCG causes transient expansion of T_regs_ (91). In a clinical trial, BCG triggers T_reg_ production in patients with type I diabetes (92), which appears to contribute to the suppression of disease and temporary restoration of islet cell function.

### Use of TNFR2 for tissue regeneration

When type 1 diabetes was first reversed in end-stage diabetic mice with boosting of TNF, the research showed an unexpected outcome (59). The pancreas of the treated diabetic mice had regenerated their islets and the original islet transplants that were performed to restore blood sugars were not needed (59). The histologic shape of the reappearing insulin secreting islets was also remarkable. The newly regenerated islets were larger in size than unaffected, untreated NOD mouse cohorts, and contrasted greatly from islets of NOD mice that had received immunosuppressive drug strategies, such as anti-lymphocyte serum or anti-CD4 or anti-CD3 antibodies, to avert diabetes (104, 105). Past autoimmune treatments of diabetic NOD mice worked almost only in pre-diabetic mice or early new-onset diabetic mice (106). Also the rescued islets of NOD mice, commonly treated with anti-CD3 immunosuppressive antibodies, were small in size, and demonstrated no or limited regeneration. The immunosuppressive drug was best administered to pre-diabetic mice or to mice with recent onset hyperglycemia. In total, this data strongly suggested that administration of TNF directly or boosting TNF indirectly with BCG or the heat-killed equivalent, CFA, had a dual mechanism of action – a direct killing of the autoreactive T cells and also a TNF effect directly on the target organ to promote healing and regeneration. Also the TNF effect on the target tissue indicated that even late stage diabetes could be reversed in large part due to the regenerative effect in contrast to a pure rescue effect, survival of existing islets without expansion, of standard immunosuppressive strategies.

The effect of TNF on the pancreas was not the only tissue showing possible regeneration with TNF stimulation. In the field of neuroregeneration, the Ting laboratory showed TNF similarly promoted proliferation of oligodendrocytes progenitors and remyelination (6). Gradually the broader literature reported the regenerative effect of TNF and TNFR2 agonism on heart regeneration, bone marrow stem cells, and even neuron regeneration in the setting of Parkinson’s disease model in mice (11, 60, 66, 107).

## Conclusion

An overlapping feature across autoimmune disorders is various defects in TNF signaling through its two receptors. TNFR2 is a more attractive molecular target than TNFR1 because of its limited cellular expression. A variety of strategies utilizing TNFR2 agonism can be pursued for treatment of autoimmune disease and also used for regenerative medicine therapies. TNFR2 agonism has been associated with selective death of autoreactive T cells in type 1 diabetes and with induction of T_regs_. It holds promise for treating other autoimmune disorders featuring dysregulation of NFkB, which is a key component of the TNFR2 signaling pathway.

## Conflict of Interest Statement

The authors declare that the research was conducted in the absence of any commercial or financial relationships that could be construed as a potential conflict of interest.
